# Mucopenetrating Janus Nanoparticles For Field-Coverage Oral Cancer Chemoprevention

**DOI:** 10.1007/s11095-022-03465-x

**Published:** 2023-01-12

**Authors:** Nahal Habibi, Caroline Bissonnette, Ping Pei, Daren Wang, Albert Chang, Jeffery E. Raymond, Joerg Lahann, Susan R. Mallery

**Affiliations:** 1 Biointerfaces Institute, Departments of Chemical Engineering, Material Science and Engineering, Biomedical Engineering, and Macromolecular Science and Engineering, University of Michigan, 2800 Plymouth Rd, Ann Arbor, MI 48105 USA; 2 Division of Oral Maxillofacial Pathology, College of Dentistry, The Ohio State University, 305 W. 12th Ave, Columbus, OH 43210 USA; 3 Department of Stomatology, Faculty of Dentistry, University of Montreal, Montreal, QC Canada; 4 The Ohio State University Comprehensive Cancer, 460 W. 10th Avenue, Columbus, OH 43210 USA

**Keywords:** chemoprevention, controlled-release preparations, nanoparticle drug delivery system, oral cancer, tocilizumab

## Abstract

**Introduction:**

Oral squamous cell carcinoma (OSCC), is associated with high morbidity and mortality. Preemptive interventions have been postulated to provide superior therapeutic options, but their implementation has been restricted by the availability of broadly applicable local delivery systems.

**Methods:**

We address this challenge by engineering a delivery vehicle, Janus nanoparticles (JNP), that combine the dual mucoadhesive properties of a first cationic chitosan compartment with a second hydrophobic poly(lactide-co-glycolide) release compartment. JNP are designed to avoid rapid mucus clearance while ensuring stable loading and controlled release of the IL-6 receptor antagonist, tocilizumab (TCZ).

**Results:**

The JNP featured defined and monodispersed sizes with an average diameter of 327 nm and a PDI of 0.245, high circularities above 0.90 and supported controlled release of TCZ and effective internalization by oral keratinocytes. TCZ released from JNP retained its biological activity and effectively reduced both, soluble and membrane-bound IL-6Rα (71% and 50%). In full-thickness oral mucosal explants, 76% of the JNP breached the stratum corneum and in 41% were observed in the basal cell layer indicating excellent mucopenetrating properties. When tested in an aggressive OSCC xenograft model, TCZ-loaded JNP showed high levels of xenograft inhibition and outperformed all control groups with respect to inhibition of tumor cell proliferation, reduction in tumor size and reduced expression of the proto-oncogene ERG.

**Conclusion:**

By combining critically required, yet orthogonal properties within the same nanoparticle design, the JNP in this study, demonstrate promise as precision delivery platforms for intraoral field-coverage chemoprevention, a vastly under-researched area of high clinical importance.

**Supplementary Information:**

The online version contains supplementary material available at 10.1007/s11095-022-03465-x.

## Introduction

Nanoparticles have transformed therapeutic delivery and enabled novel treatment modalities for cancer treatment. Oral cancer is a highly debilitating disease that dramatically impacts the lives of patients. Notably, oral squamous cell carcinoma (OSCC) is among the most challenging-to-treat human cancers. Clinical issues are elevated by the insidious nature of its early disease and the reliance upon radical surgery as the primary treatment modality [1, 2]. Even if patients achieve a surgical cure, they are still destined to suffer significant morbidity due to loss of vital facial structures essential for eating, speaking and esthetics. Similar to many carcinomas, OSCC does not occur *de novo*, but arises from a precursor surface epithelial lesion termed oral intraepithelial neoplasia (OIN). While not all OIN lesions progress to OSCC, up to one-third recur following complete, microscopically-confirmed excision, and up to 87% of high risk lesions (WHO-based binary grading system) progress to OSCC [3]. In addition, persons suffering from DNA repair deficits, e.g., Fanconi anemia (FA), are highly susceptible to field cancerization and OSCC [4]. Finally, there is a patient cohort, often with social histories negative for established OSCC risk factors, e.g., tobacco use or oncogenic human papillomaviruses (HPV) that develop multifocal premalignant lesions (proliferative verrucous leukoplakia) throughout the oral cavity [5].

Chemoprevention is the use of natural or synthetic compounds to induce regression or prevent progression of premalignant disease [6]. Ideal chemopreventive agents are highly effective and nontoxic with established mechanisms of action. In contrast, standard chemotherapy treats established cancers, employs cytotoxic agents that target rapidly dividing cells, e.g., tumor, bone marrow, GI tract, and results in deleterious side effects. While chemoprevention is clearly the optimal approach from patient-centric and cost-efficacy perspectives, poor chemopreventive agent bioavailability and the failure to achieve therapeutically-relevant intraoral levels from systemic delivery have limited the success of OSCC chemoprevention trials [7]. Notably, the pharmacologic advantages of local delivery formulations, such as the Janus nanoparticles (JNP) discussed in this study, may circumvent these previous shortcomings [8, 9].

Due to its pro-inflammatory, pro-angiogenic and pro-proliferative effects, IL-6 is a key cytokine in the development of many cancers including OSCC [10]. In addition, levels of IL-6 are often elevated in the sera and saliva of patients with OIN lesions, which support the importance of IL-6 even prior to overt OSCC development [11, 12]. Our labs have shown that OSCC cells release appreciable levels of IL-6 in addition to IL-6R, thereby establishing the potential for autocrine-paracrine growth loops even in IL-6R negative cells [13, 14]. Consequently, local injections of the IL-6R antagonist, tocilizumab (TCZ), significantly suppressed growth of OSCC xenografts [14].

It is thus plausible that a therapeutically effective, localized strategy to provide nontoxic, effective, chemopreventive coverage throughout the entire mouth is needed to address current clinical problems. The oral mucosa, however, presents major challenges for local transmucosal drug delivery systems. Mucus is a viscoelastic and adhesive hydrogel that employs highly crosslinked and entangled fibers to trap and eliminate xenobiotics and protect the underlying epithelium. In addition, the glycosylated segments within the mucin fiber hydrophobic domains demonstrate high affinity for positively charged molecules while the mucus network interacts with hydrophobic particles [15]. An effective nanoparticle formulation will need to penetrate the mucus to reach the underlying epithelium, and evade clearance by intraepithelial Langerhans cells [16, 17]. To avoid rapid mucus clearance mechanism and to ensure delivery of therapeutic cargos to the underlying, proliferative epithelium, the engineered drug delivery system must overcome the mucus and surface keratin and penetrate through the epithelium.

Nanocarriers possess unique properties making them desirable candidates for mucosal drug delivery. In addition to the protection of the drug and minimizing off-target side effects, mucoadhesive and mucopenetrating nanoparticles can prolong the contact time of the formulation with oral mucosa and facilitate delivery to the underlying cells [18]. A variety of materials, including polymers, lipids, inorganic carriers, polymeric hydrogels and biomolecular scaffolds as well as device configurations such as, cylindrical implants, thin films, and microspheres have been successfully employed to increase therapeutic efficacy by controlled drug delivery formulations [19–21]. Due to their unique properties that include large surface to volume ratio and the capacity to bind, absorb and transport therapeutic agents, nanoscale formulations have emerged as a promising drug delivery approach. In addition, the small size (range 1- ~ 300 nm) of nanoparticles is amenable to cell internalization *via* passive diffusion or endocytosis [22, 23]. Treatment selectivity can also be enhanced by strategies such as decoration with molecules that bind to overexpressed target cell antigens or use of agents to augment target cell absorption [22].

Mucoadhesive polymers can enhance the retention time of the drug leading to improved drug penetration, localization and efficacy [24]. The transit time of mucoadhesive systems bound to mucin is determined by the physiological turnover time of the mucus layer [25]. Overcoming the mucus barrier and achieving longer retention time in the cell surface requires a nanoparticle formulation that can efficiently penetrate through the mucus barrier and accumulate in the underlying epithelium [15, 25]. For the mucoadherent drug delivery formulation to be able to further penetrate through the mucus barrier, the surface charge of the system is known to play a major role [26]. Previous reports have shown that penetration through the mucosa can be improved effectively by neutral particles presenting high density of both positively and negatively charged groups on their surface [26–29]. This feature is incorporated into the design of PLGA-chitosan JNP by the presentation of positively charged chitosan compartment side-by-side of negatively charged PLGA compartment resulting in a net neutral surface charge (zeta potential of -1.5 mV ± 0.5).

By virtue of their two morphologically anisotropic and distinct compartments that enable concurrent delivery of two chemically distinct compounds, Janus nanoparticles are both structurally and functionally distinct from standard nanoparticles [29, 30]. During malignant transformation, OIN lesions undergo multiple growth-enabling molecular and biochemical perturbations [3]. The concurrent use of chemopreventives with complementary mechanisms of action, such as would occur with JNP delivery, provides a strategy to address this concern [14]. Previous studies from our labs have demonstrated combined use of tocilizumab with the synthetic vitamin A derivative, fenretinide, enhances chemopreventive efficacy in an OSCC xenograft model [14]. Furthermore, we have determined that fenretinide retains its bioavailability and chemopreventive activity following sustained release from polylactide-co-glycolide millicylinders [21]. These data directed the current JNP design i.e. for concurrent delivery of tocilizumab and fenretinide which will be contained within the chitosan and PLGA compartments, respectively.

Electrohydrodynamic (EHD) co-jetting, a methodology that utilizes laminar flow of two streams in parallel capillary needles without convective mixing, has been shown to be effective in creating JNP [31]. Due to rapid solvent evaporation, the initial flow-determined arrangement of the input polymers will be mirrored in the resulting bicompartmental nanoparticles [32, 33]. EHD co-jetting has been used to fabricate carriers for delivering cancer drugs, siRNA, and imaging agents in different compartments [31, 34–36]. Based on the importance of IL-6 in OSCC development, the purpose of this study is to formulate, characterize and evaluate TCZ-loaded JNP *in vitro* and *in vivo* for OSCC chemoprevention.

## Materials and methods

### Electrohydrodynamic Co-Jetting of PLGA-Chitosan JNP

The electrohydrodynamic co-jetting technique, which entails two parallel 26G needles as capillaries, was used to create the JNP in accordance with methodology in use in our lab [37–41]. Briefly, two polymeric solutions comprised of 1) 1.3 w/v%, 5:2 w/w glycol chitosan: Poly (ethylene glycol) diglycidyl ether in 1:1 v/v% DI water: Ethylene glycol and 2) 1 w/v% PLGA in dimethylformamide, were pumped at a rate sufficient to create a laminar flow. After application of voltage and Taylor cone formation, the JNP were electrosprayed onto a collector sheet followed by maintaining the particles under vacuum for ≥ 1 week. JNP underwent serial centrifugation to enable size selection, then characterized by Dynamic light scattering (DLS) and Nanosight Nanoparticles Tracking Analysis (NTA) (Malvern Panalytical, UK).

### JNP Characterization via Scanning Electron Microscopy (SEM) and Dynamic/Electrophoretic Light Scattering (DLS/ELS)

SEM images were obtained using a FEI Nova 200 Nanolab SEM/FIB at the Michigan Center for Materials Engineering at acceleration voltages of 5 kV, then processed using ImageJ (Wayne Rasband, NIH, > 500 JNP/sample) to obtain the respective JNP size distribution. A Zetasizer Nano ZS (Malvern Panalytical) was employed for DLS/ELS measurements of the JNP’ particle size and zeta potential, respectively. Final particle size and zeta potential were the average of triplicate measurements.

### TCZ Encapsulation and Assessment of Bioactive TCZ Release from JNP

TCZ was incorporated into the jetting solution of chitosan compartment prior to jetting, *via* substitution of the amount of deionized water previously used for empty nanoparticles. The encapsulation was done at a drug loading of (30:100) taking into account both compartments. Drug loading was calculated as the mass of the drug divided by the combined mass of the drug and polymer in both compartments. To assess TCZ release in a similar particle shipping condition, sucrose was mixed in the jetting solution at 1:360 mol ratio [42] (TCZ:sucrose) then TCZ loaded nanoparticles were collected, flash frozen, thawed, centrifuged, and resuspended in 2 ml “optimized TCZ buffer" (100 mM L-arginine hydrochloride, 10 mM L-histidine, 10 mM L-histidine hydrochloride monohydrate, 30 mM L-methionine, 150 µM Polysorbate 80, pH 6.37) + 1% sucrose to a final concentration of 7 × 10^10^ particles/mL and then placed into a rotator in the incubator at 37°C. At specific time intervals, the particle suspensions were centrifuged to pellet the particles. The supernatant was removed and analyzed to measure the amount of released TCZ. The particles were re-suspended in fresh release buffer. Timed collections of released TCZ were analyzed *via* a TCZ mAb-based ELISA (IBL, Minneapolis, MN). Total immunoreactive TCZ levels reflected cumulative release over time. The total loading of the sole nanoparticle protein, TCZ, was assessed using Pierce 660-nm protein assay according to manufacturer's instructions.

### Nanoparticle Internalization by Human Oral Keratinocytes

An oral squamous cell carcinoma cell line derived from a FA patient (“FA OSCC” cells, generous gift from Dr. Susanna Wells) and HPV E6/E7 transduced human normal oral epithelial cells (ScienCell Research Labs, Carlsbad, CA “EPI” cells) were cultured in Advanced DMEM supplemented with 1X Glutamax and 5% heat-inactivated FBS (GIBCO; Life Technologies; "complete” medium). Cell lines were authenticated via short tandem repeat analyses conducted by Johns Hopkins Genetic Resources Core Facility. Cells were seeded at 2.1 × 10^4^ cells in a µ-Slide (ibidi, Gräfelfing, Germany) then incubated (37^O^C, 5% CO_2_) with PLGA-chitosan JNP suspensions (final concentration of 1.67 × 10^9^ JNP/mL, 1 × 10^8^ JNP per channel). Cells without JNP, cells without JNP and primary antibody, and JNP alone represented the experimental controls. Monolayer cells were co-incubated with JNP for 1, 3, and 18 h, then formaldehyde fixed. All cells, except the primary antibody control, were stained with the Anti-LAMP1 primary antibody (Abcam, Cambridge, MA) followed by a cocktail of secondary antibodies i.e. Goat Anti-Rabbit IgG H&L Alexa Fluor 555 (Abcam, Cambridge, MA), Phalloidin Alexa Fluor 647, followed by the nuclear stain DAPI (Thermo Fisher Scientific, Waltham, MA). Four fluorescent channels of a FV3000 confocal microscope (Olympus Life Sciences, Waltham MA) were employed. The composite images were captured with the Olympus FV3000 RS fluoview software (Olympus Life Sciences, Waltham, MA).

### Assessment of Nanoparticle Migration Through Surface Epithelium in Human Oral Mucosal Explants

Following IRB approval (OSU IRB: 2018C0077), human oral mucosal samples from consented participants undergoing elective dental procedures were obtained. Mucosal samples, patient demographics and social history data were immediately coded, and stored securely. Promptly after excision, the mucosal explants were placed in 6-well plates, followed by coating with a sample size-specific volume of the fluorescent-tagged PLGA-chitosan JNP suspension (Average size: 344 nm, 1.4 E11 JNP/ml), and incubated. During incubation (3 h, 37°C, 5% CO_2_), JNP-explants were covered with covered by a Nexcare™ Tegaderm™ film (EM, Maplewood, MN). Following incubation, tissues were embedded in O.C.T. compound (Fisher Scientific, Waltham, MA), snap frozen with liquid N_2_ cooled isopentane, covered in foil, and frozen at -80°C freezer until cryostat sectioning. Control specimens without nanoparticles were also included. JNP localization studies employed fluorescent light (FITC channel) using the Olympus BX51 fluorescence microscope (Olympus, Center Valley, PA). Images were captured using a Nikon DS-Fi1 color digital microscope camera (Nikon, Melville, NY). Depth of JNP penetration into the surface epithelium was qualitatively assessed at 400 × using a layered stratification system: basal 1/3, middle 1/3, superficial 1/3 of the epithelium, and stratum corneum as previously reported by our lab [43].

### Fluorescent Activated Cell Sorting (FACS) Quantitative Assessment of Nanoparticle Internalization

Cells were plated in serum free DMEM, incubated for 1, 3, and 18 h with fluorescent-labeled PLGA-chitosan JNP (Supplemental Table 1), followed by removal of medium and PBS × 2 rinses. Cells were dissociated (StemPro Accutase, ThermoFisher, Waltham, MA), rinsed with PBS + 2% FBS “FACS buffer”, centrifuged and re-suspended in FACS buffer three times followed by nuclear staining (DAPI, ThermoFisher, Waltham, MA). FACS control samples included DAPI stained and unstained cells with and without non-fluorescent JNP. A BD LSRFortessa™ cell analyzer (BD Biosciences, San Jose, CA) was employed on forward and side scatter parameters to create a histogram with the log of fluorescent intensity on the horizontal and side scatter area (SSC-A) on the vertical axes, respectively. Co-expression of JNP fluorescence and DAPI-stained nuclei indicated the JNP-associated cell population.

### Determination of TCZ’s Effects on Immunoreactivity of hIL-6R

The capacity of JNP-released TCZ to suppress IL-6Rα immunoreactivity was evaluated using an hL-6Rα alpha DuoSet ELISA (R&D Systems, Minneapolis, MN). Concurrent assessments with stock TCZ (ACTEMRA, Genentech, San Francisco, CA) were conducted. Conditioned medium from a pleural effusion transformed histiocytic cell line (U937 cells, ATCC CRL 1593.2), which generated high levels of sIL-6Rα (pg/cell number), was used as an additional positive control to assess TCZ’s capacity to suppress IL-6Rα immunoreactivity. The *in vitro* studies (pretreat U937 cells) employed: 1.0 µg/mL of TCZ (ACTEMRA), 10 µg/mL of TCZ (ACTEMRA), or 1.0 µg/mL of JNP-released TCZ. Of note, 7.85 µg/mL of TCZ was the maximum reproducible level released from JNP among different lots. Control wells (no treatment) were included and all wells were incubated at 37ºC for 24 h. Impact was assessed by measuring percent reduction of sIL-6R and IL-6R levels compared to control (no treatment) and was adjusted to account for differences in cell densities measured at 24 h.

### Evaluation of JNP-Released TCZ in an OSCC Tumor Regression Model

A tumor regression model, which entailed subcutaneous flank injection of an STR-validated, highly tumorigenic OSCC cell line (https://www.atcc.org/products/all/CRL-2095.aspx, SCC2095sc, 10^6^ cells suspended in 100 µl Matrigel (Corning Life Sciences, Corning, NY)) was used to assess impact of TCZ on OSCC explant growth. These studies entailed 3 experimental groups (JNP-drug free control (JNP-CTR), TCZ bolus injection and JNP-TCZ), n = 9 mice per group, bilateral flank 2095sc injections. The mice and their tumor injection sites were monitored daily with measurements recorded (calipers length x width) every 3^rd^ day. OSCC tumors developed in ~ 90% of the 2095sc-Matrigel injection sites. Treatments (q4 days, 3 total) were initiated on the 15^th^ day after tumor cell injection and consisted of: JNP-CTR, JNP-TCZ (1.2 µg final TCZ release, shipped frozen and thawed just prior to injection), and TCZ bolus (ACTEMRA, 1.2 µg). JNP suspensions and TCZ stock drug dilution employed TCZ optimized buffer + 1% sucrose. Visible tumor growth was apparent in the majority of mice by 15 days. Twenty-seven days following tumor cell injection, gross final tumor measurements were obtained and mice were sacrificed. Excised tumors were fixed in formalin for 8 h then transferred to PBS until histologic processing. Final histologic size was determined by measuring the greatest area (width x height) for each tumor. Mitotic activity was determined by the average of 10 high power fields (HPF) counted in three different areas of each tumor with the investigator blinded. Vascular density was assessed *via* immunohistochemical (IHC) staining with the immunohistochemical endothelial marker, ERG, followed by image analysis quantification (Image Pro Premier, Cybernetics, Rockville, MD). The presence of intratumoral macrophages was evaluated *via* IHC staining with the murine macrophage antibody (Antimacrophage monoclonal Ab ab56297, Abcam, Cambridge, MA) followed by image analysis of whole slide images. Select tumors, seen primarily in the JNP-TCZ mice, which were too small for triplicate counts or excessively fragmented with large Matrigel pools, were not suitable for analyses. Select photomicrographs were captured using the Leica DM750 microscope and Leica ICC50W camera (Leica, Wetzlar, Germany).

### Statistical Analyses

**Fig. 1:**
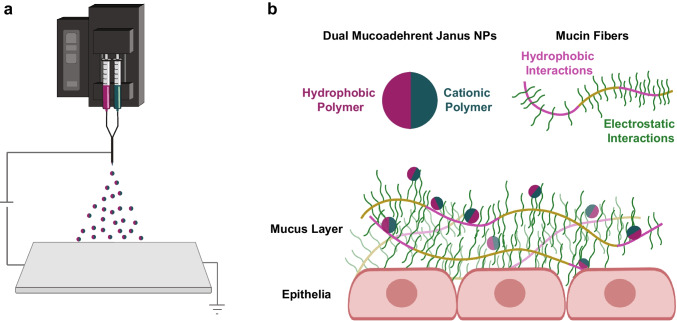
*Schematic of dual mucoadhesive PLGA-chitosan JNP and their interactions with mucosal barrier.* **(a)** Illustration of EHD co-jetting of PLGA-chitosan JNP. **(b)** Design of PLGA-chitosan JNP and dual adhesion mechanisms with mucus layer.

Data distribution, analyzed *via* a Shapiro Wilk normality test, was used to determine whether a parametric or nonparametric analysis would be employed (GraphPad Prism, San Diego, CA). The ability of TCZ to functionally impact IL-6R ELISA reactivity was evaluated by a Kruskal Wallis ANOVA followed by a Dunn’s multiple comparison test. A one-way ANOVA, followed by a Tukey’s post hoc test, were used to assess treatment effects on histologic tumor measurements, mitotic activity, and ERG expression (Fig. 1).

## Results and Discussion

### Design and Characterization of PLGA-chitosan JNP

**Fig. 2:**
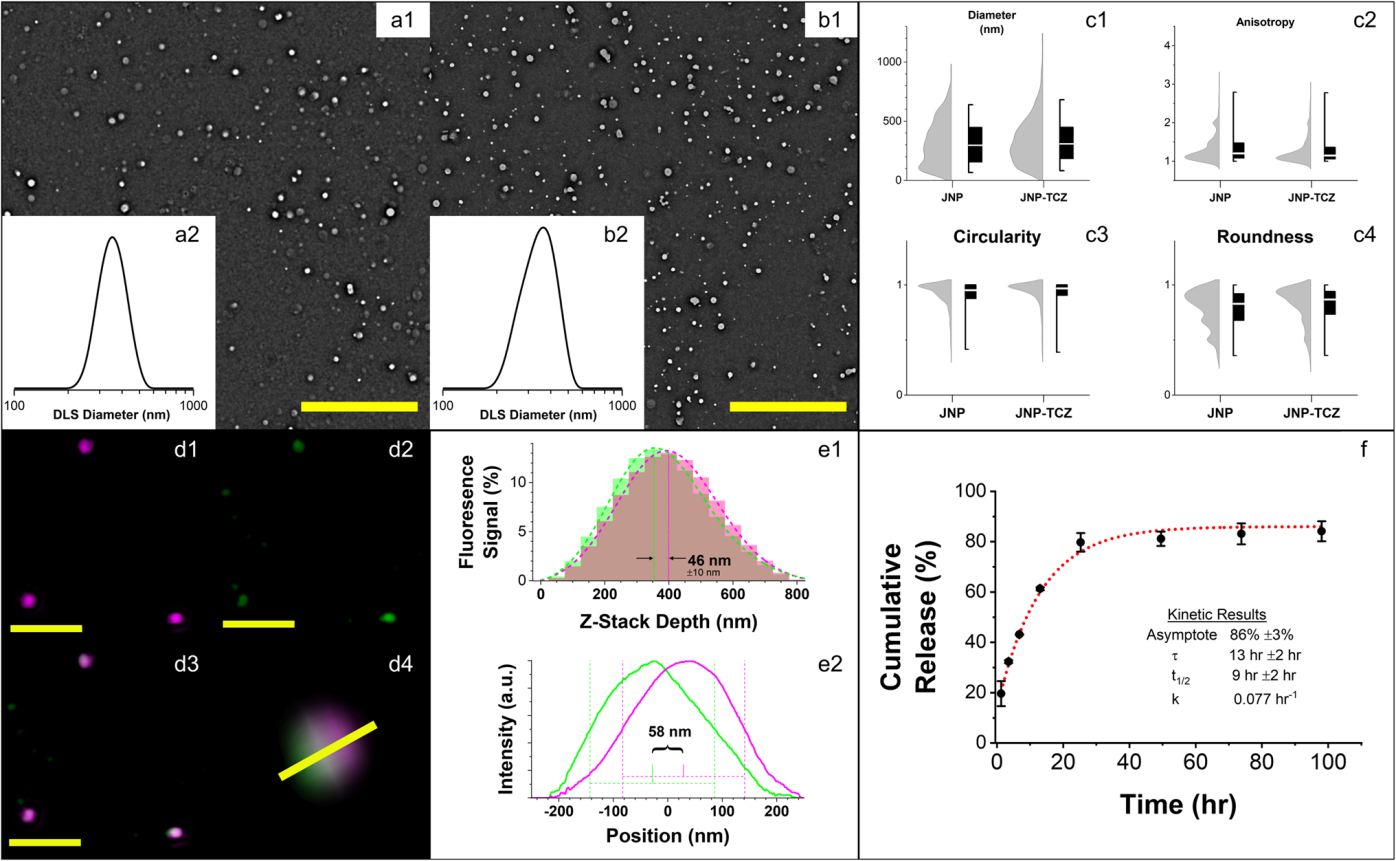
*Characterization of PLGA-chitosan JNP and JNP-TCZ.* (**a.1**) SEM images of PLGA-chitosan JNP and (**b.1**) JNP-TCZ; scale bar is 10 μm; insets of intensity-average DLS (**a.2**, **b.2**). (**c.1**–**c.4**) Key geometric factors of PLGA-chitosan JNP and JNP-TCZ based on SEM analysis (left: half-violin of histogram; right: median as line, box as IQR, and whiskers as 1/99 percentiles). **(d.1–d.3)** Z-stack projections of SIM micrographs expressing AF488 emission (green), Nile red emission (magenta) and composite images of JNPs; scale bar of 2 μm. (d.4) 3D reconstruction of Z-projection of the JNP oriented to present the compartmental interface; field of view of 2 μm. **(e.1)** Z-stack intensity profile for both channels and **(e.2) **intensity profile of the 3D projection as indicated by profile bar in D4. **(f) **Release kinetics of active TCZ from JNP-TCZ as measured by ELISA (single exponential decay, k = 0.077%/hr).

In this study, PLGA-chitosan JNP were fabricated *via* EHD co-jetting using two parallel solutions. The first solution was comprised of PLGA dissolved at a 1% (w/v) concentration in dimethylformamide. The second solution contained glycol chitosan and poly(ethylene glycol) diglycidyl ether (PEGDE) at a 1.3% (w/v) concentration in a solvent mixture of water and ethylene glycol at 1:1 (v/v)% ratio. The compartment comprised of chitosan was chemically crosslinked using PEGDE linker molecule at a 5:2 w/w glycol chitosan / PEGD ratio to achieve stable nanoparticles. The morphology and size distribution of PLGA-chitosan JNP were obtained by SEM and further ImageJ analysis. (Fig. 2A.1, 2C). The as-prepared PLGA-chitosan JNP had an average diameter of 316 nm (Q1/Med./Q3 = 157/296/446) with PDI_SEM_ of 0.268, high circularity (Avg. = 0.91, Q1/Med./Q3 = 0.88/0.95/1.00), relatively low anisotropy (Avg. = 1.35, Q1/Med./Q3 = 1.09/1.20/1.47), and relatively high roundness (Avg. = 0.78, Q1/Med./Q3 = 0.68/0.83/0.92) based on SEM analysis of as-jetted PLGA-chitosan JNP. All nanoparticles were collected and centrifuged to separate the JNP with a target average size of 360 nm to ensure optimal cell uptake. The particles were analyzed with dynamic light scattering (DLS; Fig. 2A.2) to determine their size distribution and were successfully separated using serial centrifugation to obtain a population of JNP with an average hydrodynamic diameter of 362 nm. The slight increase in size by DLS can be attributed to a convolution of hydrodynamic effects and swelling of the JNP nanoparticles in solution. The JNP nanoparticles were assessed by dual-channel SIM (structured illumination microscopy; Fig. 2D) to confirm the bicompartmental nature of the system. Subsequent assessment of the center of each compartments signal from a Z-stack perspective (Fig. 2E.1; ca. 46 nm spacing) and a projection (Fig. 2E.2; ca. 58 nm spacing) and were found to be reasonably positioned given the size and shape of the particles.

### PLGA-chitosan JNP preserve TCZ’s immunoreactivity after release

A controlled release, local delivery formulation of the humanized monoclonal antibody that competitively inhibits the entire IL-6R complex, TCZ, was achieved via a JNP delivery platform. The chitosan compartment was selected for TCZ loading and transport due to solvent compatibility. To encapsulate TCZ in nanoparticles, the TCZ was mixed with the jetting formulation of the chitosan compartment. The TCZ-loaded PLGA-chitosan JNP (JNP-TCZ) were further characterized by SEM (Fig. 2B.1) to assess their size distribution and morphology (Fig. 2C). The fabricated JNP-TCZ had an average diameter of 327 nm (Q1/Med./Q3 = 185/295/437) with a PDI_SEM_ of 0.245. There was no significant difference between the size distribution of the PLGA-chitosan JNP and the JNP-TCZ. The circularity of JNP-TCZ (Avg. = 0.92, Q1/Med./Q3 = 0.90/0.97/1.00), however, was slightly higher than that of PLGA-chitosan JNP (*P* < 0.05). The JNP-TCZ possessed higher roundness (Avg. = 0.82, Q1/Med./Q3 = 0.74/0.87/0.94), and lower anisotropy (Avg. = 1.29, Q1/Med./Q3 = 1.06/1.15/1.35) (*P* < 0.0001). DLS was used to determine the size distribution of collected particles after serial centrifugation to isolate the 360 nm size population (Fig. 2B.2). The average hydrodynamic diameter of JNP-TCZ was determined to be 364 nm. These findings, which confirmed that the encapsulation of TCZ did not influence the average diameters and size distributions of JNP in solution, are consistent with their geometrical properties in the dry state size.

The release profile of TCZ from JNP was measured by a TCZ monoclonal antibody (mAb)-based ELISA. The retention of TCZ immunoreactivity confirmed that the structural integrity of the ELISA-immunoreactive component of the TCZ molecule loaded within the chitosan compartment was retained during the co-jetting process (Fig. 2F). In addition, the cumulative release over time showed that 84% of the encapsulated TCZ was released after four days from PLGA-chitosan JNP. Single exponential fitting was performed, providing a half-life of 9 h ± 2 h (with an asymptote of 86%, reasonable given a final data point of 84%). Due to the ease of local delivery dosing to the oral cavity, replenishment of TCZ-JNP every 5 days or sooner would be straightforward and within the realm of current care paradigms. Further, the PLGA compartment could provide a controlled-release, sustained-delivery platform for complementary chemopreventives while also enhancing drug transport and oral bioavailability *in vivo* [14, 44]. As numerous, diverse molecular perturbations arise during OIN malignant transformation, reliance on a single agent for OSCC chemoprevention is not pragmatic. We have previously shown a combination of TCZ with the synthetic vitamin A derivative, fenretinide, enhances OSCC chemopreventive benefits(14). Additional formulation studies confirmed that PLGA is an excellent vehicle for local fenretinide delivery [45]. In addition, JNP are amenable to transportation via saliva throughout the mouth, a feature that is necessary for field-coverage OSCC chemoprevention.

### Human oral keratinocytes effectively internalize JNP

**Fig. 3:**
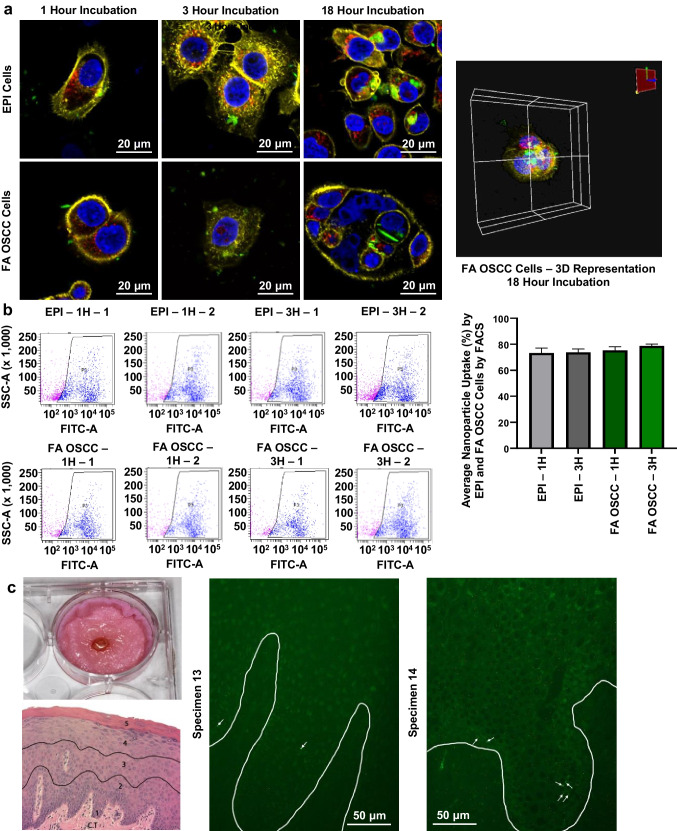
*Qualitative and quantitative assessment of PLGA-chitosan JNP internalization.* **(a)** FA OSCC and EPI cell lines incubated with JNP exhibit internalized and membrane bound PLGA-chitosan JNP. Blue – Nucleus; Red – Lysosome; Yellow – Actin; Green – JNP (40 × oil objective, 4 × zoom). **(b)** FA OSCC and EPI cell cells seeded at 1.5 × 10^4^ cells/well and incubated with 1 × 10^9^ JNP per well showed uptake ranging between 73.2% (1 h) and 78.7% (3 h). The plots depict the P3 (blue) population of cells that are the live human oral epithelial cells exhibiting dual fluorescence at 405 and 488 nm by FACS*.* **(c)** Nanoparticle penetration of clinically healthy oral gingival explants: 7 out of the 17 explants exhibited JNP (white arrows) in the basilar third. The white lines were added to highlight the epithelium-lamina propria junction.

Confocal microscopy studies confirmed that both premalignant (EPI) and FA OSCC cell lines readily internalized the fluorescent-labeled PLGA-chitosan JNP (Fig. 3a). In addition to the cytosolic location, some particles were strongly associated with external cell membranes (Fig. 3a). These experiments collectively demonstrate a positive correlation between incubation time and particle uptake with highest nanoparticle internalization at the final time point [18 h]. JNP were internalized by both EPI and FA OSCC cell lines as confirmed by colocalization of the fluorescent signals from actin filaments and nanoparticles (Fig. 3a).

Due to the unique features present in saliva i.e. high-molecular-weight mucin, pellicles and variety of hydrophobic interactions, additional investigations to assess keratinocyte florescent labelled JNP internalization during incubation in human saliva. For control purposes, these same cells lines were also incubated in PBS and Advanced DMEM medium + 5% FBS. Our data (see Supplemental Fig. 3.) demonstrate comparable uptake on fluorescent-labelled JNPs in the EPI cells line following a 3 h incubation in saliva, Advanced DMEM-5% FBS and PBS.

Complementing confocal microscopy, quantifiable fluorescence-activated cell sorting (FACS) revealed that the majority of keratinocytes in both cell lines contained membrane-bound and internalized JNP (average size 344 nm). The highest particle uptake confirmed to be cell-associated via dual positive fluorescence for NucBlue Live (viable cell nuclei) with the 488 nm fluorophore (labeled JNP) was observed in the EPI cell line (96.1% cells). The FA OSCC cells, however, still showed considerable cell uptake (86.6% cells) over 18 h (Online Resource 1). In addition, while there was a positive association between duration of exposure and particle uptake, appreciable JNP uptake was also observed at shorter time points (Fig. 3b). After a 1 h incubation, 73% and 75% particle uptake occurred in the EPI and FA OSCC cells, respectively. Importantly, these results were reproducible, with a variability of less than 14% between two separate experiments conducted with different passages of the same cell lines. Collectively, these findings confirmed human oral keratinocyte JNP internalization and strong JNP-cell membrane associations.

Depending on the drug payload and its target, e.g., receptor inhibition or modulation of intracellular signaling cascades, both locations are desirable. JNP internalization by keratinocytes may involve either endocytosis or phagocytosis. Previously, we have shown human oral keratinocyte internalization of acetylated LDL in addition to FluoSphere model nanoparticles (210 nm) [43, 46]. Mechanistic studies by Sayedyahossein *et al*. identified a vital role for integrin-linked kinases (e.g., Rac1) activation and actin polymerization during skin keratinocyte phagocytosis [47]. All of these findings recapitulate an established keratinocyte physiologic function, i.e., melanosome internalization coupled with transfer from activated melanocytes to basal layer keratinocytes during tanning or reactive oral melanosis [48].

These data are in contrast with our previous FluoSphere results that showed that the highest uptake of 18.4% (relative to histiocytic lymphoma cell line, U937) in one OSCC cell line occurred at 24 h with no detectable particle uptake after 1 h [41]. Both the FluoSpheres and the JNP were comparably-sized and possessed both negative and positive charged functional surface groups. There were, however, distinct compositional differences between FluoSpheres (biotin labeled, fluorescein-loaded polystyrene nanoparticles) relative to PLGA-chitosan nanoparticles. We note that polystyrene-associated cytotoxicity may be at least partially responsible for the observed reduced uptake of the FluoSphere model nanoparticles [20]. In contrast, PLGA nanoparticle encapsulation has been demonstrated to reduce cytotoxicity, yet retain therapeutic effectiveness of chemotherapeutic drugs [49]. While our prior studies evaluated only internalized nanoparticles, our current assays also included strongly-membrane adherent bound JNP as this cellular location is integral for TCZ’s IL-6R inhibition. Finally, as nanoparticle size affects particle uptake, our JNP (average size ~ 350 nm) were likely internalized by a caveolae-mediated endocytosis [50].

### JNP Demonstrate Surface Epithelial Penetration in Human Oral Mucosal Explants

While not all human oral mucosa is covered by a keratinized surface layer, for the JNP penetration studies we selected a rigorous model, i.e., keratinized gingival explants. Fluorescent-tagged JNP studies revealed 76% of the mucosal explants (13/17) demonstrated particle penetration past the stratum corneum while 41% (7/17) contained nanoparticles in the targeted basilar third of the epithelium (Online Resource 2). Some of the explant tissues showed particle penetration into the superficial underlying connective tissue. Control specimens showed no fluorescent particles. No specific trends regarding JNP penetration based on age, sex or clinical site were discerned among the tissue donors (Table I). Not all explants showed the same depth of nanoparticle penetration; findings that likely reflect individual differences in transport mechanisms and variation in thickness of the epithelial layers. JNP could have migrated *via* paracellular transport, which is a passive permeation process that enables small molecules to diffuse between keratinocytes, and/or energy dependent transcytosis [51, 52]. Reports have also showed that chitosan can improve paracellular permeation by temporarily disrupting the tight junctions [53, 54]. It is therefore probable that nanoparticle optimization combined with *in vivo* analyses in ATP-replete tissues will enhance nanoparticle transport to the targeted, proliferative, basal layer epithelial cells [52].

**Table I:** Participant Demographics, Donor Site, PLGA-chitosan Nanoparticle Suspension Volume Applied to the Explant and Total Nanoparticle Quantity, Time of Incubation and Maximum Depth of Penetration in the Epithelium

	Age/ Sex	Ethnicity	Smoking	Alcohol	Donor Site	Volume of Suspension	Time of Incubation	Depth of Penetration
1	72 F	Caucasian	Never-smoker	Non-drinker	Anterior maxilla	1 µL (3.79 × 10^7^ NP)	3 h	Superficial 1/3
2	59 F	Caucasian	Never-smoker	Occasional drinker	Posterior right maxilla	1 µL (3.79 × 10^7^ NP)	3 h	Stratum corneum
3a	61 F	African-American	Never-smoker	Occasional drinker	Posterior left maxilla	2 µL (7.58 × 10^7^ NP)	2.5 h	Basal 1/3
3b					Posterior left maxilla	2 µL (7.58 × 10^7^ NP)	2.5 h	Superficial 1/3
4	64 M	Caucasian	Former smoker	Occasional drinker	Posterior right mandible	4 µL (1.52 × 10^8^ NP)	3 h	Basal 1/3
5	67 F	Caucasian	Former smoker	Occasional drinker	Posterior left maxilla	4 µL (1.52 × 10^8^ NP)	3 h	Middle 1/3
6	60 F	Hispanic	Never-smoker	Non-drinker	Posterior left maxilla	2 µL (7.58 × 10^7^ NP)	3 h	Stratum corneum
7	40 M	African-American	Never smoker	Occasional drinker	Anterior maxilla	3 µL (1.13 × 10^8^ NP)	3 h	Superficial 1/3
8	69 M	African-American	Former smoker	Occasional drinker	Posterior right maxilla	4 µL (1.52 × 10^8^ NP)	3 h	Basal 1/3
9	32 F	Caucasian	Never smoker	Non-drinker	Posterior right maxilla	2 µL (7.58 × 10^7^ NP)	3 h	Superficial 1/3
10^a^	48 M	African-American	Smoker	Occasional drinker	Anterior mandible	4 µL (1.52 × 10^8^ NP)	3 h	N/A
11	63 F	Caucasian	Former smoker	Occasional drinker	Anterior maxilla	4 µL (1.52 × 10^8^ NP)	3 h	Basal 1/3
12	22 M	Caucasian	Never smoker	Occasional drinker	Anterior maxilla	3 µL (1.13 × 10^8^ NP)	3 h	Superficial 1/3
13^b^	32 F	Caucasian	Never smoker	Non-drinker	Posterior left maxilla	4 µL (1.52 × 10^8^ NP)	3 h	Basal 1/3
14a	38F	Caucasian	Never smoker	Occasional drinker	Anterior maxilla	2 µL (7.58 × 10^7^ NP)	3 h	Basal 1/3
14b					Anterior maxilla	2 µL (7.58 × 10^7^ NP)	3 h	Basal 1/3
15	60 M	Caucasian	Never smoker	Non-drinker	Posterior left maxilla	4 µL (1.52 × 10^8^ NP)	3 h	Stratum corneum
16	84 M	Caucasian	Former smoker	Occasional drinker	Posterior right maxilla	2 µL (7.58 × 10^7^ NP)	3 h	Stratum corneum

^*^Specimen 10 was Excluded Due to Poor Orientation Across Multiple Sections (No Epithelium)

^**^Specimens 8 and 13 were Collected from the Same Patient, but from Different Sites and at Different Dates

### Impact of JNP-Released TCZ on ELISA Detection of IL-6R

**Fig. 4:**
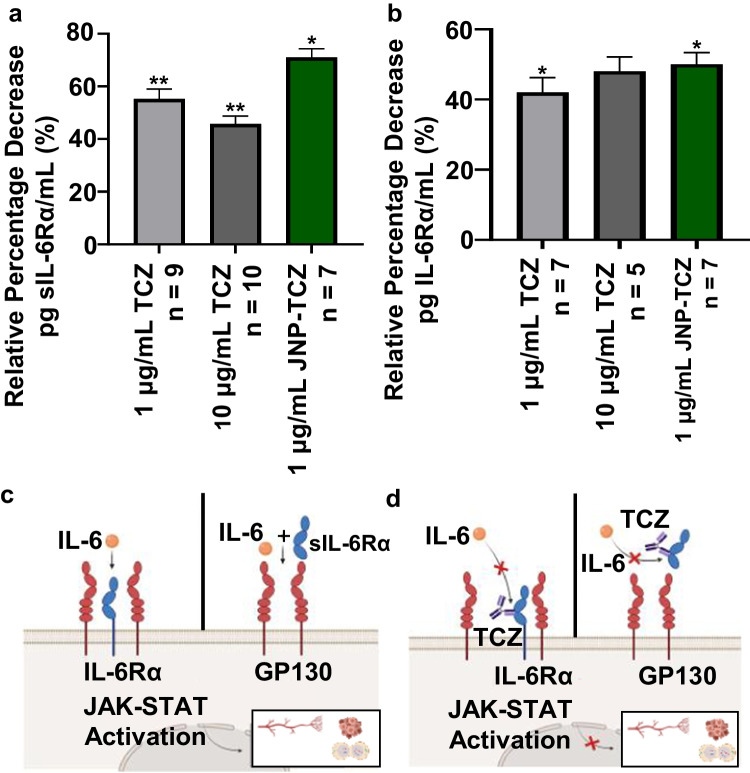
*JNP released TCZ retains bioactivity.* **(a)** ELISA data showing effects of JNP released TCZ on sIL-6R detection. Twenty-four hour treatment of U937 conditioned medium with stock TCZ at doses of 1 μg/mL (*p* < 0.01) and 10 μg/mL (*p* < 0.05) as well as 1 μg/mL (*p* < 0.05) released from JNP were associated with a significant decrease in sIL-6R secretion from U937 cells. (**b)** Similarly, TCZ treatment significantly reduced intracellular levels of IL-6R in treated U937 cells. **(c)** TCZ has shown numerous anti-tumorigenic effects through its modulation and inhibition of STAT3 via both classic (left pane) and trans-signaling (right pane). The JAK-STAT pathway favors tumor cell proliferation, angiogenesis in the tumor microenvironment, and metastasis **(d)** TCZ inhibits STAT3 phosphorylation by competitively binding to IL-6R (left pane) and sIl-6R (right pane) hence negating the downstream events. The target genes encode for proteins regulating growth and apoptosis. High concentrations of circulating IL-6 have been reported to increase resistance to both chemotherapy and radiation.

Preliminary studies confirmed that conditioned medium from the histiocytic lymphoma U937 cell line reproducibly contained high levels of sIL-6Rα (~ 350 pg/mL/1 × 10^6^ cells). Similarly, U937 cells also contained high intracellular levels of IL-6Rα (~ 500 pg/mL/1 × 10^6^ cells). Results from the hIL-6Rα ELISA revealed that both pharmaceutical dispensed TCZ and JNP-released TCZ disrupted IL-6Rα binding and therefore reduced IL-6Rα detection in U937 conditioned medium (Fig. 4a). In addition, 24 h TCZ treatment (pharmaceutical dispensed and JNP-released) of U937 cells also significantly reduced intracellular ELISA-detectable IL-6R levels (Fig. 4b). The average U937 cell viability (trypan blue exclusion, control and TCZ treated) for these studies was $\sim$ 92.5%. Collectively, the TCZ and IL-6R ELISA data confirm that JNP EHD co-jetting preserved the immunomodulatory activity of the human monoclonal antibody TCZ. The absence of complete inhibition of IL-6R may be due to the fact that the capture antibody and IL-6Rα target different TCZ epitopes. In addition, the ELISA capture antibody may overlap partially, but not completely with the TCZ-IL-6Rα complex. This premise is substantiated by preliminary competition assays performed in our lab which demonstrated failure of stock TCZ (200 mg/mL) to completely inhibit binding of the IL-6Rα reagent standard to the capture antibody. In addition, these data do not display a dose-dependent response; findings that likely reflect the 1 µg/ml dose is sufficient to provide the maximal feasible inhibition. The observed favorable performance of JNP-released TCZ relative to bolus drug delivery may reflect a slight composition difference between the JNP supernate and pharmaceutically dispensed TCZ.

### JNP-Released TCZ Retains its Bioavailability and Bioactivity and Demonstrates Significant and Superior OSCC Tumor-Regressive Effects

**Fig. 5:**
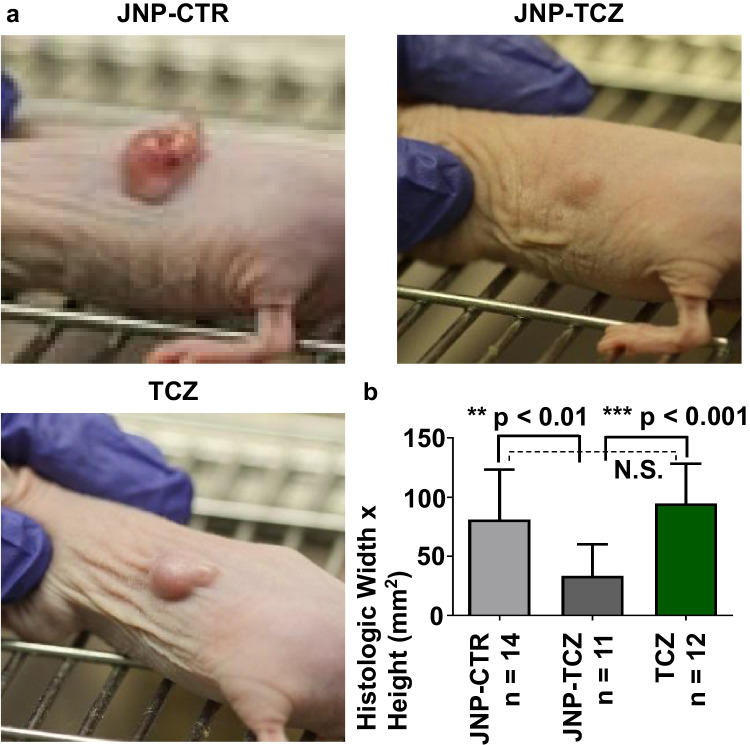
*Effects of TCZ releasing JNP on OSCC tumor induction.* **(a)** Gross OSCC tumors at day 27 prior to collection. The tumors treated with JNP-TCZ appeared clinically smaller and less erythematous. **(b)** At day 27, mice were sacrificed and tumors were excised. The latter were bisected along the greatest dimension plane and histologic size (width x height) was determined for the masses in which tumor islands were still present. The JNP-TCZ tumors were significantly smaller in size and this difference was statistically significant when comparing with JNP-CTR (p < 0.01) and TCZ tumors (p < 0.001). No significant differences were detected in comparison of the JNP-CTR and TCZ tumors. (Not Significant (N.S)).

**Fig. 6:**
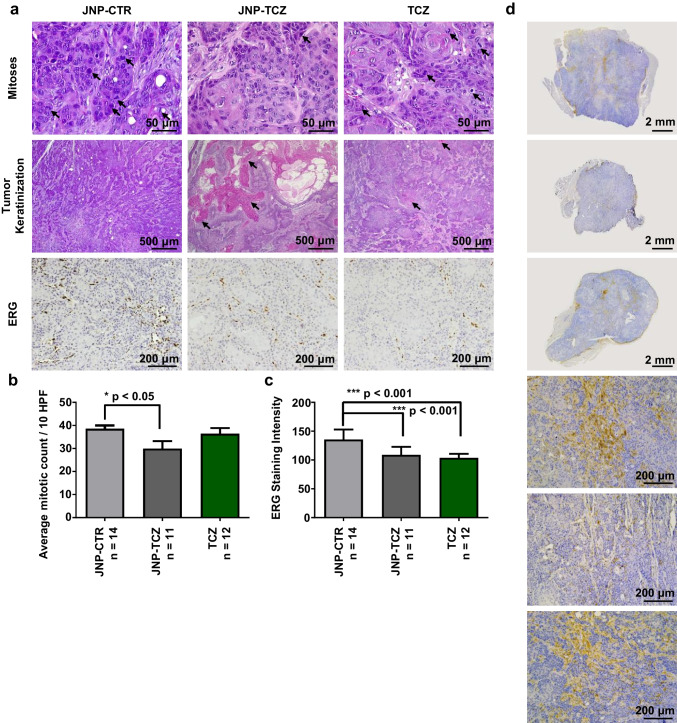
*JNP-TCZ treated OSCC tumors exhibit increased differentiation in conjunction with significantly reduced mitotic activity and vascular density.* **(a)** Mitotic figures are highlighted by black arrows, hematoxylin and eosin stain. Areas of tumor keratinization are identified with black arrows, hematoxylin and eosin stain. ERG positive cells (Brown chromogen) highlight the endothelial cells of the vascular structures in the tumors. **(b)** Treatment with JNP-TCZ led to a statistically significant reduction in mitotic activity (p < 0.05) and a decrease in vascular density (p < 0.001). Treatment with TCZ bolus injections also led to a decrease in vascular density, but the mitotic rate was similar to that measured in OSCC tumors treated with control JNP. (**c**) Murine macrophage staining. Immunohistochemical staining demonstrated murine macrophages are present in all of the tumors and primarily localized to the residual Matrigel in the tumor stroma. Overall, the CTR-JNP tumors showed the greatest number of macrophages, which were typically found in multi-macrophage aggregates. While dispersed macrophages were found in both of the TCZ treated tumors, some of the bolus TCZ tumors showed macrophage aggregates similar to those seen in control JNP tumors. Overall, the JNP-TCZ tumors showed a qualitative reduction in macrophages with only infrequent macrophage aggregates noted. These data likely reflect reduced signaling of the proinflammatory cytokine, IL-6, by TCZ in conjunction with enhanced tumor retention of adherent TCZ-JNP relative to the more readily diffusible TCZ bolus drug. Finally, as tumor-associated macrophages can facilitate tumor progression via their release of tumor promoting cytokines and immunosuppression, the observed macrophage reduction in TCZ-JNP tumors is likely chemopreventive in nature.

The OSCC tumor explants showed clinical differences that corresponded to the presence/absence of TCZ (Fig. 5a). The JNP-Control (JNP-CTR) tumors were more erythematous, findings that are consistent with preservation of the tumor microenvironment IL-6 signaling and associated inflammation and angiogenesis [55]. JNP-TCZ treatment impact on OSCC tumor growth was also clinically apparent (Fig. 5a) [14]. While one of the JNP-TCZ treated mice initially had a measurable flank mass, this mouse showed complete gross and histologic tumor regression at the final time point. Furthermore, TCZ significantly reduce tumor size only if delivered from JNP (Fig. 5b). Neither the presence of drug-free JNP, nor bolus-delivered TCZ, had an impact on tumor size. Tumor proliferation (assessed by mitotic count) aligned with microscopic tumor size as only the JNP-TCZ treated tumors showed a significant reduction in tumor cell proliferation (Fig. 6a). Furthermore, the presence of TCZ augmented tumor differentiation, as determined by increased keratin production, reduction in nuclear to cytoplasmic ratios and fewer atypical mitotic figures (Fig. 6b). These findings highlight the integral role of IL-6 in tumorigenesis, i.e., sustained tumor cell proliferation with reduced terminal differentiation [55]. In addition, as OSCC cells produce both IL-6 and sIL-6R, IL-6 likely fulfills both an autocrine and paracrine role in OSCC tumorigenesis [14]. These data also depict the benefit of sustained TCZ release *via* JNP relative to bolus delivery. Due to the JNP composition, adherence to tumor cells and stroma was likely, which could result in a local TCZ releasing reservoir. In contrast, the impact of bolus delivered TCZ would likely be transient.

Image analysis (Image Pro Premier, Media Cybernetics, Rockville, MD) quantification of the ETS-related gene (ERG) demonstrated that the presence of TCZ significantly reduced overall tumor ERG expression, regardless of delivery mechanism (Fig. 6c). These data are consistent with TCZ’s established inhibition of IL-6’s proinflammatory and proangiogenic effects. As ERG is a recognized reliable marker for endothelial cells, immunohistochemical staining for ERG was primarily employed to assess treatment effect on tumor vascularity [56]. While the majority of ERG staining was tumor vasculature-associated, some of the OSCC tumor nests also showed nuclear ERG staining. Although tumor ERG expression has been reported in other human cancers, most notably during prostate cancer development and progression, it has not been reported in OSCC [57]. Interestingly, the prospect of ERG-IL-6 crosstalk was inferred from the strong positive correlation between IL-6 and ERG levels in prostate cancer [58]. Corresponding mechanistic studies in ERG overexpressing prostate cancer cells showed concurrent upregulation of the EP2 prostanoid receptor and increased IL-6 secretion while EP2 inhibition reduced IL-6 release [58]. Although OSCC tumors express EP2, substantial OSCC tumor and cell studies are necessary to establish plausible ERG-OSCC-IL-6 associations [59]. We note, however, that given ERG’s oncogenic potential *via* its abilities to induce angiogenesis and generate tumor-promoting gene fusion products, the observed ERG reduction should convey chemopreventive benefits [57].

The presence of TCZ and JNP, combined with reduced IL-6 signaling, have the capacity to promote macrophage tumor infiltration. Intra-tumoral murine macrophages, however, were observed in all three experimental groups. While multi-macrophage aggregates were more prevalent in the control JNP and bolus TCZ tumors, these differences were not statistically significant (Fig. 6d). Although TCZ would inhibit IL-6’s proinflammatory signaling, the sustained presence of macrophages likely reflects the multitude of chemo-attractants released by the OSCC cancer cells [60].

Collectively, our chemopreventive data demonstrated that JNP-released TCZ conveyed a local therapeutic advantage; findings that likely reflect several parameters: (i) Due to the JNP composition and surface charge, adherence to tumor cells and stroma was likely, which results in a local TCZ releasing reservoir thereby augmenting chemopreventive efficacy. In contrast, the impact of bolus delivered TCZ would likely be transient. (ii) While subcutaneously injected bolus-delivered TCZ displays an absorption half-life of 2–4 days in patients, dosing levels directly impact TCZ’s elimination (61). Notably, TCZ follows nonlinear kinetics, with increased dose prolonging the drug half-life. (iii) As the injected TCZ dose administered to the nude mice was proportionately smaller (~ 50 fold) relative to the human subcutaneous dose, local drug clearance was likely more rapid and resulted in a more transient effect.

Our *in vivo* chemopreventive studies employed a challenging OSCC tumor-regression model that tested the efficacy of an agent intended for secondary and tertiary chemoprevention as a solitary chemotherapeutic agent. It is therefore essential to place these data in the context of future clinical applications. Even during the most challenging clinical role, i.e., tertiary chemoprevention, JNP-TCZ mediated tumor growth suppression and angiogenic inhibition would likely convey greater impact during incipient tumor development. Furthermore, for optimized OSCC chemopreventive efficacy, TCZ in conjunction with a mechanistically-complementary second chemopreventive would be employed.

## Conclusions

Results from this study, which demonstrate successful incorporation and sustained release of a structurally complex protein from JNPs. i.e., TCZ, confirm the potential of precision nanomedicine. The JNPs exhibited monodispersed sizes with an average diameter of 327 nm and a PDI of 0.245 and high circularities above 0.90. ELISA analyses revealed JNP-released TCZ retained its immunoreactivity and bioactivity as demonstrated by the ability to inhibit IL-6R ELISA binding. Cell-JNP internalization-uptake studies revealed over 86% of target oral keratinocytes showed JNP internalization and JNP-human oral mucosal explant analyses confirmed successful JNP penetration beyond the ingress-inhibiting stratum corneum while over 40% of the tissue explants revealed JNP presence in the basilar third of the surface epithelium. Lastly, the proof of concept *in vivo* studies confirmed JNP-released TCZ’s therapeutic efficacy, i.e., significant reductions in tumor sizes and proliferation indices were only observed in the JNP-release TCZ treatment group. With regard to future clinical applications, we have identified a second chemopreventive, fenretinide, which functions in an additive fashion with TCZ [13, 14]. In addition, incorporation of the JNP into a dispersing formulation such as a mouth rinse would enable field coverage throughout the oral cavity, which is essential for persons at high risk for field cancerization throughout the mouth. This includes persons with DNA repair deficits, e.g., Fanconi anemia or proliferative verrucous leukoplakia (PVL), which is associated with multifocal lesions with a very high rate of malignant transformation [5]. In conclusion, to our knowledge, these studies are the first to demonstrate successful encapsulation and controlled release of bioactive TCZ from a local delivery formulation.

## Supplementary Information

Below is the link to the electronic supplementary material.

Supplementary file1 (PDF 17938 KB)

## Data Availability

All data analyzed during this study are included in this published article (and its supplementary information file). Other raw data required to reproduce these findings are available from the corresponding author on request.
