# Supplementary figures and images for: Mucopenetrating Janus Nanoparticles For Field-Coverage Oral Cancer Chemoprevention
Source: Pharm Res. 2023 Jan 12;40(3):749–64. doi: 10.1007/s11095-022-03465-x (PMC10036282; doi:10.1007/s11095-022-03465-x)

**Medium**

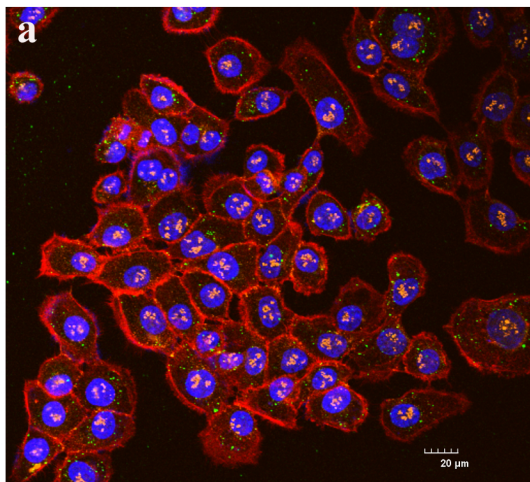

**PBS**

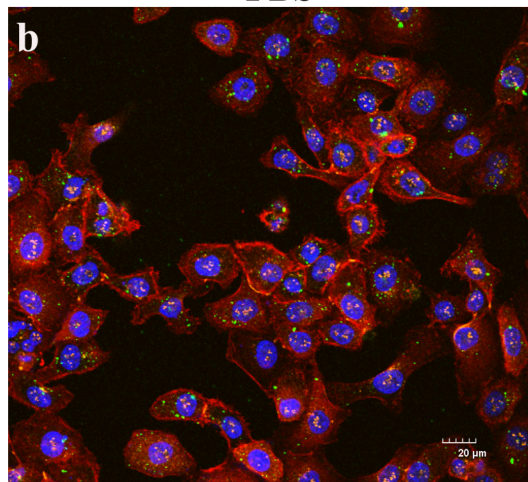

**Saliva**

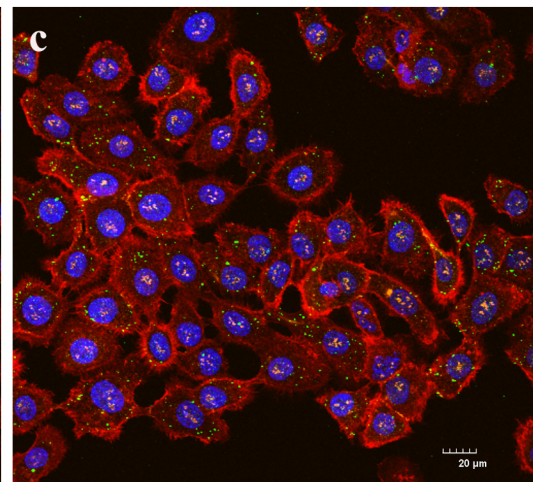

Supplement: Supplementary file 1 — Supplementary file1 (PDF 17938 KB) [file 11095_2022_3465_MOESM1_ESM.pdf]
